# Integrated Microbiomics and Metabolomics Reveal That Moisture Content and *Lactiplantibacillus plantarum* Synergistically Regulate Fermentation Quality, Microbial Community, and Metabolite Profiles of Amaranth Silage

**DOI:** 10.3390/microorganisms14061317

**Published:** 2026-06-12

**Authors:** Muqier Zhao, Jian Bao, Xiaowei Jiang, Yahong Liu, Dong Pan, La Zhu, Yuting Yan, Jiayu Zhao, Zhijun Wang, Gentu Ge

**Affiliations:** 1 College of Grassland Science, Inner Mongolia Agricultural University, Hohhot 010019, China; 2 Key Laboratory of Forage Cultivation, Processing and High Efficient Utilization, Ministry of Agriculture, Hohhot 010019, China; 3 Inner Mongolia Academy of Agricultural and Animal Husbandry Sciences, Hohhot 010031, China; 4 Institute of Grassland Research, Chinese Academy of Agricultural Sciences, Hohhot 010010, China; 5 Ulanqab Academy of Agriculture and Forestry Sciences, Ulanqab 012000, China

**Keywords:** amaranth silage, *Lactiplantibacillus plantarum*, moisture content, microbiomics, metabolomics, fermentation quality

## Abstract

This study investigated the effects of moisture content (80% vs. 70%) and *Lactiplantibacillus plantarum* inoculation on the fermentation quality, microbial community structure, and metabolite profiles of amaranth (*Amaranthus hypochondriacus*) silage using integrated microbiomics (full-length 16S rRNA sequencing) and untargeted metabolomics (UPLC-MS/MS). The results showed that high-moisture silage without inoculation (AhGCK) exhibited poor fermentation quality, characterized by high pH (5.09) and low lactic acid content (1.42% FM). Inoculation with *L. plantarum* significantly reduced pH (to 4.16) and increased lactic acid accumulation (to 3.65% FM) under high-moisture conditions. Wilting to 70% moisture combined with *L. plantarum* inoculation (AhSLP) achieved the best fermentation quality, with the lowest pH (4.20) and highest lactic acid (4.46% FM). Microbial community analysis revealed that *Enterobacter cloacae* dominated in AhGCK, whereas *L. plantarum*, *Lentilactobacillus buchneri*, and *Levilactobacillus brevis* became dominant after inoculation and wilting. Metabolomics identified 497 compounds across all treatments, with differential metabolites primarily enriched in pathways related to amino acid metabolism, carbohydrate metabolism, and biosynthesis of secondary metabolites such as diterpenoids and isoquinoline alkaloids. Highly significant correlations (*p* < 0.01) were observed between *Enterobacter* and cyclohexylammonium, between dTDP-3-O-methyl-β-L-rhamnose and 2-hydroxy-2H-benzo[h]chromene-2-carboxylate, between *Lentilactobacillus* and 3-ketosucrose (positive), and between *Limosilactobacillus* and 8-methylthiooctyl glucosinolate (positive), whereas *Lactiplantibacillus* and *Escherichia Shigella* showed no correlations with differential metabolites. These findings indicate that inoculating *Lactobacillus plantarum* at specific moisture levels (80% and 70%) promotes directed microbial community succession (as exemplified by positive correlations of *Lentilactobacillus* and *Limosilactobacillus* with beneficial metabolites) and optimized metabolite accumulation, which significantly lowers dry matter loss during fermentation and enhances the output of usable silage. This mechanism offers a practical theoretical foundation for improving amaranth silage production and boosting feed yield.

## 1. Introduction

The global livestock industry is facing increasing pressure from the growing scarcity of high-quality forage resources, making the development of high-yield, high-protein alternative forages a critical pathway for ensuring the sustainable development of the sector. Amaranth (*Amaranthus hypochondriacus*) is a C4 plant characterized by rapid growth, high biomass yield, and outstanding protein content. Its leaf crude protein content can reach 20–25% on a dry matter basis, and its composition of essential amino acids, including lysine and methionine, is superior to that of conventional gramineous crops such as maize, indicating its potential as a high-quality protein forage [1]. However, fresh amaranth typically has a moisture content of 80–85% and a low water-soluble carbohydrate content (approximately 2% on a dry matter basis). These characteristics predispose it to excessive growth of non-target microorganisms such as clostridia during ensiling, leading to butyric acid fermentation and substantial losses of dry matter and protein, which severely compromise the quality of its silage [2].

The essence of ensiling is a microbial-driven fermentation process under anaerobic conditions, in which beneficial microorganisms, such as lactic acid bacteria, convert plant water-soluble carbohydrates into organic acids, leading to a rapid decline in pH. This inhibits the activity of spoilage microorganisms and enables the long-term preservation of nutrients [3]. For unconventional protein forages such as amaranth, characterized by high moisture content (80–85%) and low water-soluble carbohydrate content (approximately 2% dry matter), the ensiling process faces even greater challenges. Moisture content is a key process parameter regulating microbial succession and metabolic pathways during ensiling. Studies have shown that when the moisture content of raw material exceeds 70%, the elevated water activity not only provides a favorable environment for competitive microorganisms such as clostridia but also alters the community assembly process by affecting cellular osmotic pressure and substrate diffusion rates, leading to the accumulation of undesirable metabolites such as butyric acid and ammonia nitrogen [4]. Through integrated microbiome and metabolome analysis of alfalfa silage with varying moisture contents, it was found that under high-moisture conditions, the relative abundance of the class Clostridia increased significantly and exhibited a strong positive correlation with metabolites such as butyric acid and putrescine [5]. In contrast, after reducing the moisture content to 65–70%, the relative abundance of lactic acid bacteria increased by 2.3-fold, and the fermentation metabolic pathways shifted toward homolactic fermentation [6]. This study revealed the underlying mechanism by which moisture content modulates niche competition among key microbial taxa, thereby reshaping the metabolic network [7].

*L. plantarum*, as a typical representative of facultative heterofermentative lactic acid bacteria, is widely recognized as a model bacterium for silage additive research due to its rapid acid production rate, broad substrate utilization spectrum, and strong environmental adaptability [8]. Studies have indicated that the addition of *L. plantarum* can inhibit the growth of spoilage microorganisms such as clostridia and Enterobacteriaceae through multiple mechanisms, primarily rapid acidification and competition for nutrients, as well as bacteriocin secretion, thereby reducing protein degradation and dry matter loss. However, the regulatory efficacy of *L. plantarum* is significantly influenced by raw material characteristics, particularly moisture content. The meta-analysis by Li et al. demonstrated that in silage systems with a moisture content exceeding 70%, the promoting effect of exogenous *L. plantarum* on lactic acid accumulation was reduced by approximately 32% compared to systems with a moisture content below 70%, indicating that high-moisture conditions may weaken the competitive advantage of *L. plantarum* [9]. Therefore, elucidating the interaction between moisture content and *L. plantarum* is of great significance for optimizing the processing technology of amaranth silage.

In recent years, the maturation of microbiomics and metabolomics technologies has provided powerful tools for revealing the micro-level mechanisms of silage fermentation. High-throughput sequencing (e.g., full-length 16S rRNA sequencing) enables precise tracking of microbial community dynamics at the species level during ensiling, while untargeted metabolomics (e.g., LC-MS/MS) allows comprehensive profiling of metabolite accumulation patterns, thereby elucidating the intrinsic logic of fermentation regulation from an integrated perspective of microbial community, metabolites, and fermentation quality [10,11]. Although previous studies have demonstrated the synergistic regulatory effects of moisture content and Lactobacillus plantarum inoculation in corn, alfalfa, and sorghum silages [4,11], different forage materials may exhibit unique response patterns due to variations in their physicochemical properties and epiphytic microbial communities.

Amaranth, as a highly promising high-protein forage resource, holds great significance in alleviating the shortage of high-quality protein feed [1]. However, research on amaranth silage is still in its early stages. Existing studies have primarily focused on changes in conventional indicators of fermentation quality, lacking a systematic analysis of the interaction between moisture content and *L. plantarum* from the perspective of microbial and metabolic synergy. In particular, the unique chemical composition of amaranth, high protein, low sugar, and high moisture, suggests that its patterns of microbial community succession and metabolic responses during ensiling may differ from those of typical forages such as maize and alfalfa. Therefore, how the interaction between moisture content and *L. plantarum* affects the microbial community structure and metabolite composition of amaranth remains poorly understood at the systematic omics level [12]. Based on the aforementioned research background and the gaps in existing studies, this study proposes the following core hypothesis: in the amaranth silage system, the interaction between moisture content and *L. plantarum* co-determines the formation of fermentation quality and metabolite profiles by synergistically regulating the niche construction of microbial communities and the remodeling of metabolic functions. Specifically, different levels of moisture content may selectively enrich or inhibit specific microbial taxa by altering water activity, substrate availability, and microenvironmental osmotic pressure, thereby guiding the differential development of fermentation pathways. Meanwhile, exogenous addition of *L. plantarum* accelerates the establishment of homolactic fermentation under suitable moisture conditions, suppresses the competition of spoilage microorganisms such as clostridia, and influences the accumulation patterns of final metabolites through the regulation of key metabolic pathways. To validate this hypothesis, this study uses mature grain amaranth as the experimental material, sets different moisture content gradients, and establishes treatments with and without *L. plantarum* inoculation under each moisture condition. A systematic investigation will be conducted using a simulated ensiling system, aiming to provide a theoretical basis for the processing and utilization of high-protein forages such as amaranth.

## 2. Materials and Methods

### 2.1. Materials and Ensilage Preparation

The test material was *Amaranthus hypochondriacus* (cultivar ‘K472’), with seeds provided by Jilin Zhongxian Ecological Agriculture Technology Co., Ltd. (Jilin City, China) The experiment was sown on 19 June 2023 at the Hailiutu Science and Technology Park of Inner Mongolia Agricultural University (40°41′30″ N, 111°2′30″ E). The experimental area has a temperate continental monsoon climate with an average annual frost-free period of 133 days. Sowing was performed manually in rows at a depth of 2 cm, with a row spacing of 20 cm and a plant spacing of 15 cm. The seeding rate was 9 kg·hm^−2^, resulting in a plant density of 1.5 × 10^5^ plants·hm^−2^. During the growing period, irrigation was applied according to soil moisture, and regular manual weeding was carried out without the use of herbicides or fungicides. Uniform water and fertilizer management, manual weeding, and pest control were implemented throughout the experiment to ensure normal growth of amaranth. Harvesting was conducted on 20 September 2023, at the milk-ripe stage. Six independent 1 m^2^ quadrats were randomly selected in the field, and whole plants were harvested from each quadrat. Each quadrat served as a true biological replicate (rather than a subsample from a composite bulk sample). After harvest, the plants from each quadrat were placed separately on clean plastic sheeting, thoroughly mixed individually, and subsampled using the quartering method for each replicate. A portion of the fresh sample from each quadrat was immediately used to determine the initial chemical composition and microbial counts of that replicate; the remaining portion of each quadrat was used for moisture adjustment and independent silage preparation. A total of six such biological replicates were established per treatment. Based on the initially measured moisture content of the fresh sample, the material was adjusted to 80% and 70% moisture by adding distilled water or natural air drying, designated as the GFM group (80%) and SFM group (70%), respectively. After adjustment, the moisture content was re-checked to confirm the target values. Subsequently, the material from both groups was cut into approximately 2–3 cm segments using a handheld chipping cutter for silage preparation.

Amaranth materials with moisture contents adjusted to 80% and 70% were used for silage preparation, and four treatment groups were established. For the material with 80% moisture content, the treatment groups were designated as the additive-free control (AhGCK) and the group inoculated with *L. plantarum* JYLP-002 (AhGLP). For the material with 70% moisture content, the treatment groups were designated as the additive-free control (AhSCK) and the group inoculated with the same *L. plantarum* strain (AhSLP). *L. plantarum* JYLP-002 (patent No. 201810629862.9; provided by Shandong Zhongke Jiayi Bioengineering Co., Ltd., Qingzhou City, China; deposited at the China General Microbiological Culture Collection Center under accession No. CGMCC 15801 on 23 May 2018) was applied at a dose of 1 × 10^6^ CFU per gram of fresh matter (FM). The additive was dissolved in deionized water, and the solution was uniformly sprayed using a handheld sprayer at a rate of 10 mL per kilogram of amaranth; the control silages received an equal amount of deionized water. Subsequently, 500 g of amaranth samples were placed into polyethylene bags, vacuum-sealed, and stored for 60 days before sampling and analysis.

### 2.2. Nutritional Composition and Fermentation Product Analyses

Amaranth was harvested at maturity and subjected to two moisture content levels (80% and 70%) and two treatment groups (CK and LP). After 60 days of ensiling, samples were collected to evaluate fermentation performance, microbial community composition, and metabolite profiles. The dry matter (DM) content was determined for the amaranth material which was dried for 72 h at a constant temperature of 65 °C with a forced-air oven. The dried amaranth material was ground using a 1 mm screen (FW100, Taisite Instrument Co. Ltd., Tianjin, China) for nutritional compositions analysis. The Association of Official Analytical Chemists method was used to determine the crude protein (CP) content [13]. Fiber fractions, such as acid detergent fiber (ADF) and neutral detergent fiber (NDF), were analyzed according to the method [14]. The method with a colorimetric reaction with anthrone reagent was used to determine the water-soluble carbohydrate (WSC) content [15].

The chopped 10 g amaranth was sampled and homogenized with 90 mL deionized water in a glassware graduated cylinder. The resulting extract was filtered through cleaner gauze (4 layers) of previously rinsed cheesecloth to ensure clarity. The pH value of the amaranth silage was measured by the extracted filtrate using a portable pH meter, with the probe previously calibrated for accuracy. The filtered extract was then analyzed for organic acid levels, including lactic acid (LA), acetic acid (AA), propionic acid (PA), and butyric acid (BA), using high-performance liquid chromatography (HPLC) as described by You et al. [16]. Ammonia-nitrogen (NH_3_-N) content was determined following the phenol-hypochlorite method reported previously by Kleinschmit et al. [17].

### 2.3. Microbiological Analysis

Microbial populations including LAB, yeasts, mold, anerobic bacteria, and coliform bacteria in the FM and silage were assessed as described in a previous report [16]. After 60 days of fermentation, bacterial community composition of amaranth silage was characterized via 16S rRNA gene sequencing. Total DNA was extracted following [18], and PCR amplification of the 16S rRNA gene was performed using primers 27F/1492R under the conditions described by Guo et al. [19]: 98 °C for 2 min; 30 cycles of 98 °C for 30 s, 50 °C for 30 s, and 72 °C for 60 s; and a final extension at 72 °C for 5 min. Each treatment had six replicates. Purified PCR products were sequenced on the Pacbio_SMRT platform (Biomarker Technologies, Beijing, China).

Alpha diversity indices (coverage, Chao1, ACE, Simpson, Shannon) were calculated using QIIME v1.9.1 and Mothur v.1.30. Principal coordinate analysis (PCoA) based on UniFrac distances was conducted in R v3.2.5. OTUs were classified using RDP Classifier v2.2 against the SILVA database (Release 128) with a 0.7 confidence threshold and taxonomically assigned at phylum, genus, and species levels. Correlation heatmaps were generated using R-based tools. LEfSe analysis was employed to identify differential biomarkers between groups [20]. Microbial functions were predicted using PICRUSt2 based on the KEGG database, inferring functional gene composition by aligning sequences with phylogenetic references and integrating IMG genomic data [21].

### 2.4. Determination of Metabolites

Metabolite extraction and detection were performed using ultra-high performance liquid chromatography-mass spectrometry (UPLC-MS/MS): 50 mg of amaranth sample was accurately weighed and mixed with 1000 μL of extraction solution (methanol: acetonitrile: water = 2:2:1, *v*/*v*/*v*) containing an internal standard (2 mg/L), vortexed for 30 s, then homogenized with ceramic beads at 45 Hz for 10 min, followed by ultrasonication in an ice-water bath at 4 °C for 10 min. The sample was kept at −20 °C for 1 h and centrifuged at 12,000 rpm for 15 min; 500 μL of the supernatant was dried under vacuum. The dried metabolites were reconstituted in 160 μL of reconstitution solution (acetonitrile: water = 1:1, *v*/*v*), vortexed, sonicated, and centrifuged, after which 120 μL of the supernatant was transferred to a vial for analysis. A quality control (QC) sample was prepared by pooling 10 μL from each sample for instrument stability monitoring. Raw data were processed through peak detection, alignment, noise filtering, and normalization to obtain a metabolite expression matrix. Orthogonal partial least squares discriminant analysis (OPLS-DA) was used to evaluate differences between groups. The criteria for identifying differential metabolites were: variable importance in projection (VIP) > 1.0 derived from the OPLS-DA model, fold change (FC) ≥ 2 or ≤ 0.5 (i.e., |log_2_FC| ≥ 1), and a *p*-value (two-tailed Student’s *t*-test) adjusted by false discovery rate (FDR) < 0.05. Metabolites meeting all three criteria were considered statistically significant differential metabolites. For metabolites with VIP > 1.0 and |log_2_FC| ≥ 1 but an FDR slightly above 0.05, their contribution to the OPLS-DA model and biological relevance were used for auxiliary judgment.

### 2.5. Data Analysis

Experimental data were initially organized and preprocessed using Microsoft Office Excel 2019. For the characteristics of the raw amaranth material prior to ensiling, one-way analysis of variance (ANOVA) was performed to assess differences between moisture levels. For all post-ensiling data, including fermentation quality indicators, microbial community profiles, and metabolomic features, a two-way ANOVA was conducted using SAS software (version 9.4; SAS Institute Inc., Cary, NC, USA) to evaluate the main effects of moisture content, *L. plantarum* inoculation, and their interaction. A total of six biological replicates were analyzed for each treatment group. Subsequently, Tukey’s honest significant difference (HSD) test was used for multiple comparisons, and significant differences among treatment means were indicated by different letters (*p* < 0.05). Data visualization and analysis were conducted using Origin 2021 software. For microbiome and metabolome datasets, corresponding R packages were used for analysis and visualization. Specifically, metabolomic data were processed using Student’s *t*-test and fold-change analysis. Preliminary screening of differential metabolites between treatment groups was based on variable importance in projection (VIP) scores derived from orthogonal partial least squares discriminant analysis (OPLS-DA) models, followed by further filtering using *p*-values or false discovery rate (FDR) from univariate analysis.

## 3. Results

### 3.1. Chemical Composition and Microbial Counts of Fresh Amaranth

The chemical composition and microbial counts of fresh samples of amaranth with different moisture content gradients are shown in Table 1. The DM content was 21.00% and 32.03%, respectively, which showed significant differences (*p* < 0.05). The DM content was inversely proportional to the CP and WSC contents, whereby the lower the DM content, in turn, was associated with lower CP and WSC contents. However, high DM content was associated with higher NDF and ADF content.

### 3.2. Chemical Composition and Fermentation Quality of Silages

The chemical composition and fermentation quality of silages with or without *L. plantarum* with different moisture content gradients are shown in Table 2. The moisture content had no significant effect (*p* > 0.05) on NDF, ADF, WSC, PA content, pH, and NH_3_-N/TN, and a highly significant effect (*p* < 0.01) on DM, CP, LA and AA content. The DM, CP and LA contents of AhSCK and AhSLP were significantly higher than those of AhGCK and AhGLP (*p* < 0.05), the AA contents of AhSCK were significantly higher than AhGCK (*p* < 0.05). There were significant or highly significant effects (*p* < 0.01 or 0.05) on LA content and pH of silage with or without additives, and no significant effects (*p* > 0.05) on other indicators. The pH and LA contents of the treatment groups without additives at different moisture contents were significantly higher and lower than those with additives (*p* < 0.05), the interaction of moisture content and additives had significant and highly significant effects on the pH and LA contents of silage (*p* < 0.05 and *p* < 0.01).

### 3.3. Microbial Community Diversity and Composition

**Figure 1:**
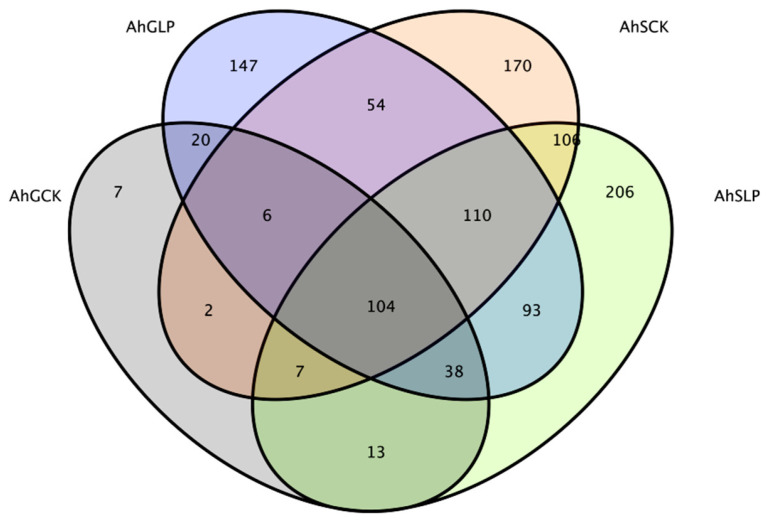
Venn diagram showing shared and unique OTUs among the four treatment groups. Note: Each ellipse represents one treatment group. The overlapping regions indicate the number of OTUs common to the corresponding groups, while non-overlapping regions represent OTUs unique to a single group. AhGCK: high moisture (80%) without inoculation; AhGLP: high moisture with *L. plantarum* inoculation; AhSCK: low moisture (70%) without inoculation; AhSLP: low moisture with *L. plantarum* inoculation. Each ellipse represents one treatment group: green for AhGCK, red for AhGLP, blue for AhSCK, and orange for AhSLP.

The Venn diagram can show the number of common and unique features between samples and visualize feature overlap. Combined with species represented by features, common microorganisms in different samples can be identified. As shown in Figure 1, the total number of species in four groups was 104. In high-moisture silage groups, AhGCK and AhGLP had seven and 147 unique species respectively, and 20 common species. In the low-moisture silage treatment group, AhSCK and AhSLP had 170 and 206 unique species respectively and 106 common species. AhGCK and AhSCK shared two species, and AhGLP and AhSLP shared 93 species.

**Figure 2:**
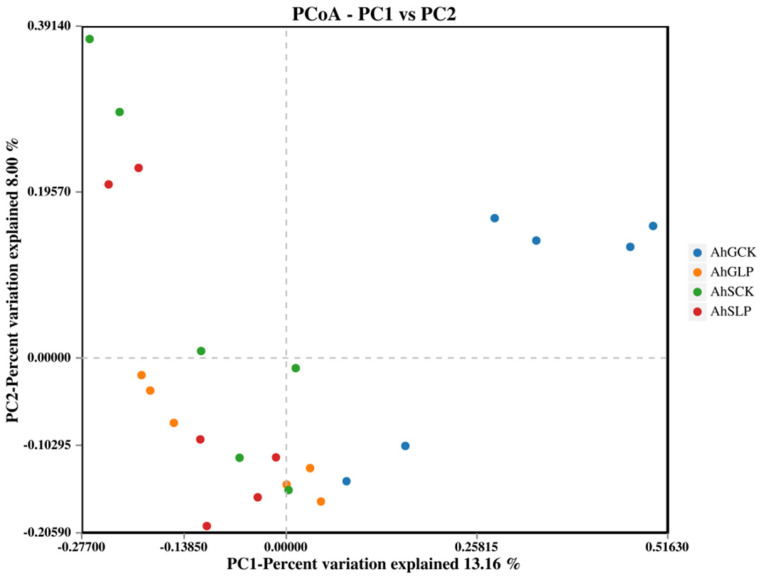
Principal coordinate analysis of bacterial communities based on weighted UniFrac distances. Note: Each point represents an individual sample. Different colors denote different treatment groups. The percentages on the *x*- and *y*-axes indicate the contribution of the first and second principal components to the total variance among samples, respectively.

Table 3 shows the alpha diversity analysis of different treatments. Moisture had no significant effect on the silage alpha diversity index (*p* > 0.05), while there was a significant difference between additives on the silage Shannon index (*p* < 0.05). The interaction of moisture and additives significantly affected the ACE, Chao1 index, and coverage (*p* < 0.05). Under the same moisture conditions, the Chao1 index of AhGLP was significantly higher than that of AhGCK (*p* < 0.05), and the Simpson and Shannon indices of AhSLP were significantly higher than those of AhSCK (*p* < 0.05). In additive-free treatments with different moisture contents, the Chao1 index of AhGCK was significantly lower than that of AhSCK (*p* < 0.05). The coverage was higher than 99% for all treatments. The PCoA enables the classification of multiple samples to show the differences in species diversity between different groups (Figure 2). The distance between AhGCK and the other three groups was greater, indicating larger flora variability. However, the closer distance among the other three treatments suggested no significant difference in their flora.

**Figure 3:**
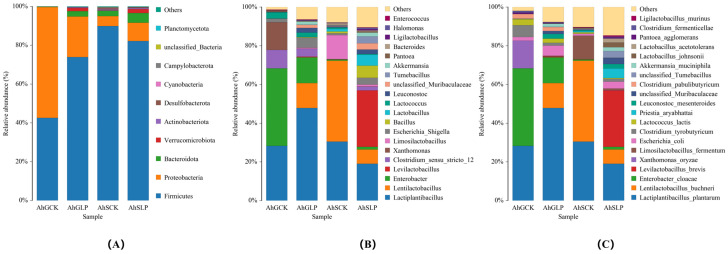
Relative abundance of bacterial taxa at the phylum, genus, and species levels in amaranth silage. Note: (**A**) Phylum level (top 10). (**B**) Genus level (top 20). (**C**) Species level (top 20). The horizontal axis shows sample groups, and the vertical axis shows relative abundance as a percentage. Different colors represent different taxonomic groups.

In high-moisture silage without inoculation (AhGCK), the relative abundance of Firmicutes was significantly lower than in low-moisture silage without inoculation (AhSCK), whereas Proteobacteria showed the opposite trend. A similar pattern was observed between inoculated groups: AhGLP (high moisture) had lower Firmicutes and higher Proteobacteria than AhSLP (low moisture). Notably, the Firmicutes abundance in AhGLP was even lower than that in AhSCK, indicating that moisture reduction had a stronger effect on promoting Firmicutes than inoculation did. The relative abundance of species at the genus level (top 20) for silages with different moisture content is shown in Figure 3B. After the anaerobic fermentation process, the dominant genus of fermenting silage with different moisture contents were different, among which the dominant genus of fermentation process in AhGCK were *Lactiplantibacillus*, *Enterobacter*, and *Xanthomonas*, and the dominant genus in AhGLP were *Lactiplantibacillus*, *Lentilactobacillus*, and *Enterobacter*. The dominant genus in AhSCK were *Lactiplantibacillus*, *Lentilactobacillus*, and *Limosilactobacillus*. The dominant genus in AhSLP were *Lactiplantibacillus*, *Levilactobacillus*, and *Lentilactobacillus*.

The dominant species differed markedly among treatments. In AhGCK, *L. plantarum*, *Enterobacter cloacae*, and *Xanthomonas oryzae* prevailed, with *E. cloacae* and *X. oryzae* being characteristic of high-moisture, non-inoculated silage. By contrast, low-moisture silage without inoculation (AhSCK) was dominated by *L. plantarum*, *Lentilactobacillus buchneri*, and *Limosilactobacillus fermentum*, with *L. buchneri taking the place of the Enterobacterium*. Inoculation with *L. plantarum* altered the species profile: AhGLP (high moisture + inoculation) was dominated by *L. plantarum*, *L. buchneri*, and *Enterobacter cloacae*, while AhSLP (low moisture + inoculation) was dominated by *L. plantarum*, *Levilactobacillus brevis*, and *L. buchneri*. Thus, the key distinction between the two inoculated groups was the presence of *Levilactobacillus brevis* in low-moisture silage (AhSLP), which was absent in high-moisture inoculated silage (AhGLP). Moreover, *X. oryzae* was abundant only in AhGCK and absent in all other groups.

### 3.4. Correlations Between Fermentation Quality Indicators and Bacterial Communities

**Figure 4:**
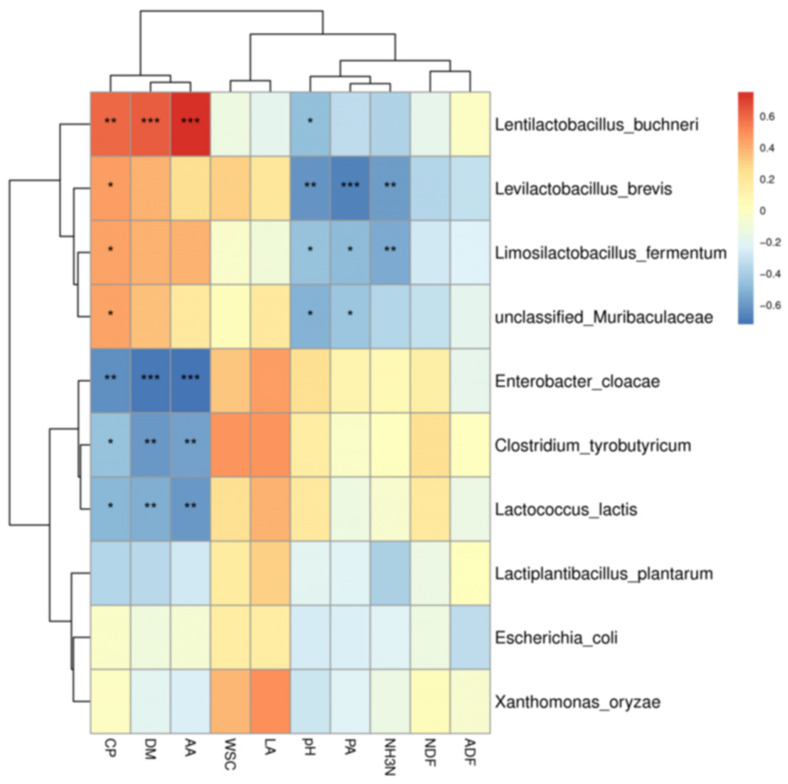
Correlation heatmap between fermentation quality indicators and the top 10 bacterial genera. Note: Each cell represents the Spearman correlation coefficient between a quality indicator and a bacterial genus. Red indicates positive correlation and blue indicates negative correlation. * indicates *p* < 0.05, ** indicates *p* < 0.01, and *** indicates *p* < 0.001.

The heat map of correlations between silage quality indicators and bacterial communities is shown (top 10) in Figure 4. CP, DM and AA content were negatively correlated with *Lactococcus lactis*, *Clostridium tyrobutyricum*, and *Enterobacter cloacae* (*p* < 0.05) while they were significantly positively correlated with *L. buchneri* which showed a significant positive correlation (*p* < 0.05). In addition, *Levilactobacillus brevis*, *Limosilactobacillus fermentum*, and *unclassified Muribaculaceae* were significantly positively correlated with CP content (*p* < 0.05). *Levilactobacillus brevis* and *unclassified Muribaculaceae* were significantly negatively correlated with pH and PA (*p* < 0.05) in the same way that *L. buchneri* was negatively correlated with pH (*p* < 0.05).

### 3.5. Metabolomic Profiling and Differential Metabolites

**Figure 5:**
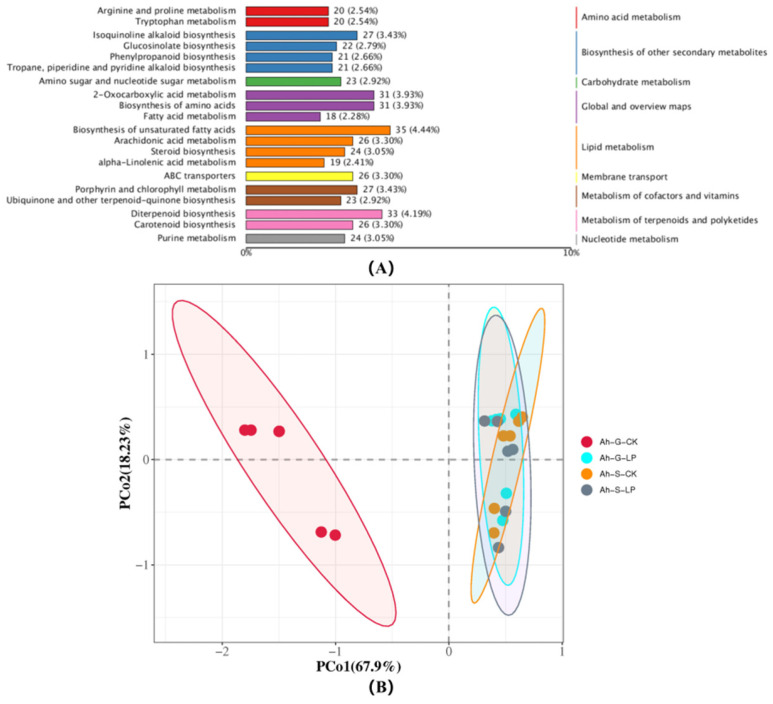
Principal coordinate analysis (PCoA) of metabolite profiles among the four treatments. Note: (**A**) Distribution of identified metabolites across KEGG pathways. The numbers and percentages indicate the count and proportion of metabolites annotated to each pathway (top 20 shown). (**B**) Principal coordinate analysis (PCoA) of metabolite profiles based on Bray-Curtis dissimilarity. Each point represents an individual biological replicate (*n* = 6 per group). Different colors indicate different groups: green, AhGCK (80% moisture, without *L. plantarum*); red, AhGLP (80% moisture, with *L. plantarum*); blue, AhSCK (70% moisture, without *L. plantarum*); orange, AhSLP (70% moisture, with *L. plantarum*). QC samples (pooled quality controls, gray triangles) are also shown to demonstrate analytical repeatability. The percentages on the *x*- and *y*-axes represent the variance explained by the first principal component (87.9%) and the second principal component, respectively.

A total of 497 compounds were identified in the four groups, distributed among amino acid metabolism, biosynthesis of other secondary metabolites, carbohydrate metabolism, global and overview maps, lipid metabolism, membrane transport, metabolism of cofactors and vitamins, metabolism of terpenoids and polyketides, and nucleotide metabolism (Figure 5A).

**Figure 6:**
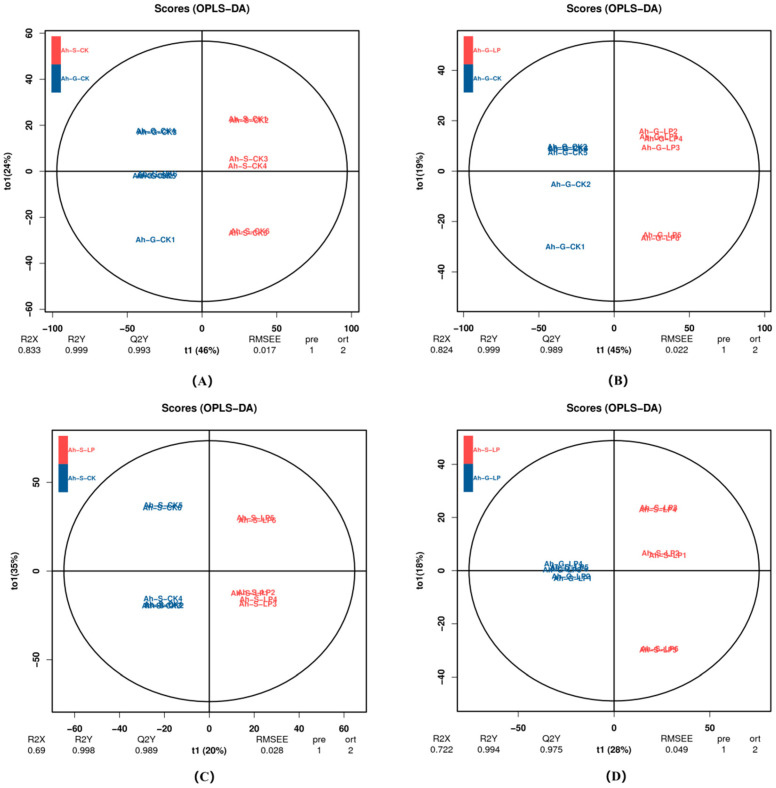
Orthogonal partial least squares discriminant analysis score plots for pairwise comparisons between treatment groups. Note: (**A**) AhGCK vs. AhSCK; (**B**) AhGCK vs. AhGLP; (**C**) AhSCK vs. AhSLP; (**D**) AhGLP vs. AhSLP. Each point represents a sample. R2Y and Q2 values indicate model goodness-of-fit and predictive ability, respectively.

**Figure 7:**
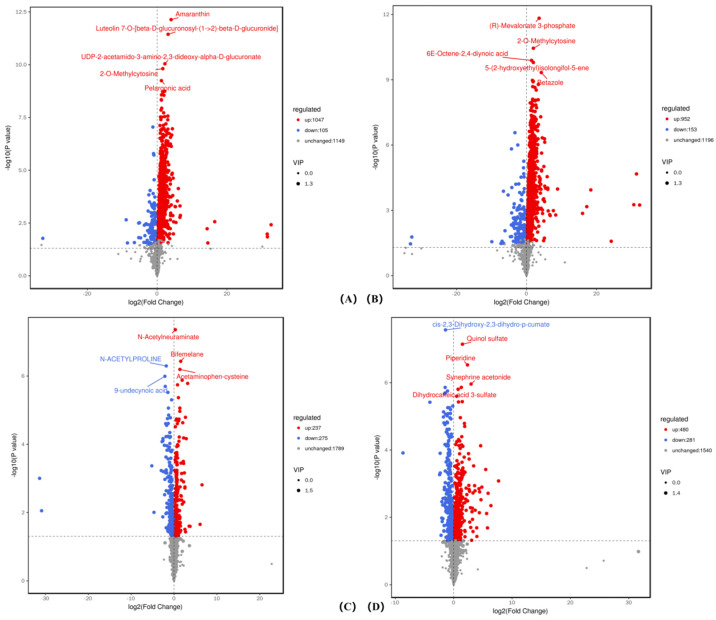
Volcano plots of differentially abundant metabolites for each pairwise comparison. Note: (**A**) AhGCK vs. AhSCK; (**B**) AhGCK vs. AhGLP; (**C**) AhSCK vs. AhSLP; (**D**) AhGLP vs. AhSLP. Each point represents a metabolite. Red points indicate significantly up-regulated metabolites (|log_2_(fold change)| ≥ 1 and *p* < 0.05), blue points indicate significantly down-regulated metabolites (|log_2_(fold change)| ≤ −1 and *p* < 0.05), and gray points indicate non-significant metabolites. The *x*-axis shows log_2_(fold change) (positive values: up-regulated in the first-named group; negative values: up-regulated in the second-named group). The *y*-axis shows -log_10_ (*p*-value) from Student’s *t*-test (two-tailed), where higher values indicate greater statistical significance. The size of each point is proportional to the variable importance in projection (VIP) score derived from the OPLS-DA model (VIP > 1.0 used for preliminary selection). The top five metabolites with the smallest *p*-values are labeled with their names (when available).

PCoA analysis is shown in Figure 5B. AhGCK, AhGLP, AhSCK, and AhSLP were separated, confirming experimental data accuracy and reliability. AhSCK differed significantly from the other three groups and was distant in the graph, with high component repeatability. QC samples overlapped, indicating high data reliability for further testing. OPLS—DA, a multivariate statistical analysis method for discriminant analysis, can assist in marker metabolite screening by calculating VIP, which measures metabolite expression pattern strength and explanatory power for sample group discrimination. R^2^X, R^2^Y, and Q^2^ are used to evaluate the OPLS—DA model, with values closer to 1 indicating a more reliable model. Samples under different treatments split on both sides, showing significant metabolic differences. Analysis of OPLS—DA evaluation model parameters showed R^2^Y > 0.99 and Q^2^ > 0.97 between different treatment samples, indicating a stable and reliable OPLS-DA model (Figure 6). The Volcano Plot offers a quick overview of metabolite content differences between two groups and their statistical significance. The differential expression Volcano Plot is shown in Figure 7. Results indicate that the AhGCK_vs_AhSCK comparison group had 1152 differential metabolites (DAMs) (1047 up-regulated, 105 down-regulated). The AhGCK_vs_AhGLP comparison group had 1105 DAMs (952 up-regulated, 153 down-regulated). The AhSCK_vs_AhSLP comparison group had 512 DAMs (237 up-regulated, 275 down-regulated), and the AhGLP_vs_AhSLP comparison group had 761 DAMs (237 up-regulated, 281 down-regulated).

**Figure 8:**
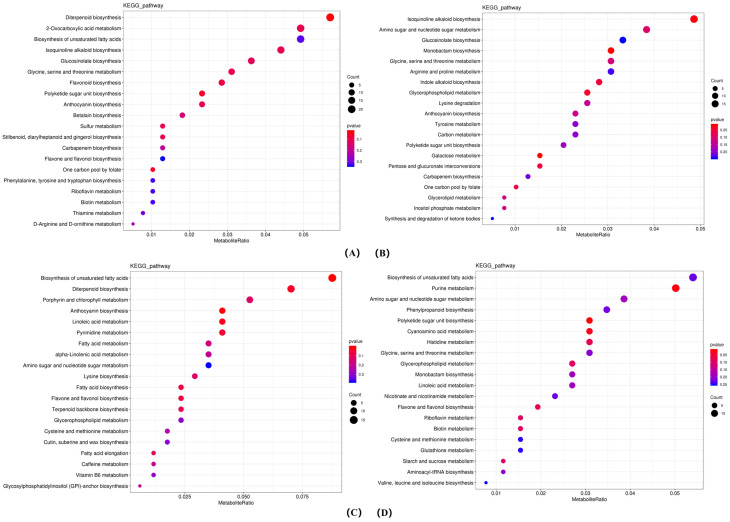
KEGG pathway enrichment analysis of differentially abundant metabolites. Note: (**A**) AhGCK vs. AhSCK; (**B**) AhGCK vs. AhGLP; (**C**) AhSCK vs. AhSLP; (**D**) AhGLP vs. AhSLP. Each bubble plot displays the top 20 enriched KEGG pathways (or all significant pathways, depending on data) for the respective comparison. The *x*-axis represents the rich factor (the ratio of the number of differentially abundant metabolites (DAMs) annotated to a given pathway to the total number of metabolites annotated to that pathway in the KEGG database). The *y*-axis lists the pathway names. The color of each bubble indicates the adjusted *p*-value (e.g., Benjamini-Hochberg false discovery rate, FDR) ranging from red (lower *p*-value, more significant) to blue (higher *p*-value). The size of each bubble is proportional to the number of DAMs enriched in that pathway. Pathways with FDR < 0.05 (or *p* < 0.05) were considered significantly enriched. Where appropriate, only the most significantly enriched pathways are labeled for clarity.

As shown in Figure 8, KEGG enrichment analysis of the differential metabolites revealed that a total of 180 DAMs were enriched in 20 KEGG pathways in the AhGCK_vs_AhSCK comparison group, mainly in diterpenoid biosynthesis, 2-Oxocarboxylic acid metabolism and biosynthesis of unsaturated fatty acids. In the AhGCK_vs_AhGLP comparison group, a total of 178 DAMs were enriched in 20 KEGG pathways, mainly in isoquinoline alkaloid biosynthesis, and in the AhSCK_vs_AhSLP comparison group, 111 DAMs were enriched in the biosynthesis of unsaturated fatty acids and diterpenoid biosynthesis pathways. In the AhGLP_vs_AhSLP comparison group, a total of 140 DAMs were enriched in KEGG pathways such as biosynthesis of unsaturated fatty acids and purine metabolism.

### 3.6. Integrated Correlation Analysis Between Differential Metabolites and Differential Microbial Genera

**Figure 9:**
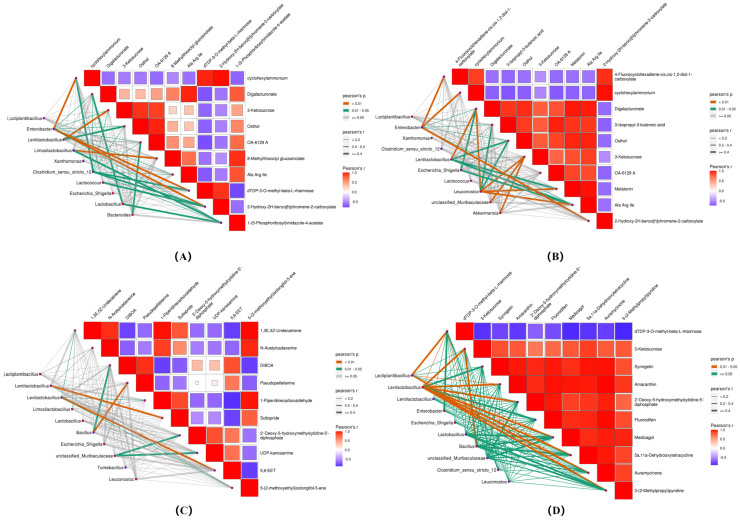
Correlation network heatmaps between differentially abundant metabolites and differential microbial genera. Note: (**A**) AhGCK vs. AhSCK; (**B**) AhGCK vs. AhGLP; (**C**) AhSCK vs. AhSLP; (**D**) AhGLP vs. AhSLP. Each cell represents the correlation coefficient between a metabolite (row) and a bacterial genus (column). Red indicates positive correlation and blue indicates negative correlation. *p* < 0.05 was considered significant; *p* < 0.01 was considered highly significant.

The correlations between the differential metabolites and differential flora were analyzed using the top 10 frequencies and the results are shown in Figure 9. Figure 9A shows the correlation between the differential metabolites for the AhGCK_vs_AhSCK comparator group. The results showed that Cyclohexylammonium did not correlate with dTDP-3-O-Methyl-beta-L-rhamnose and 2-Hydroxy-2H-benzo[h]chromene-2-carboxylate (*p* > 0.05) and showed significant negative correlations with the other seven differential metabolites (*p* < 0.05). Digalacturonate, 3-ketosucrose, Osthol, OA, 6129 A and 8-Methylthiooctyl glucosinolate were negatively correlated with dTDP-3-O-Methyl-beta-L-rhamnose and 2-Hydroxy-2H-benzo[h] chromene-2-carboxylate showed a significant negative correlation (*p* < 0.05). However, dTDP-3-O-Methyl-beta-L-rhamnose and 2-Hydroxy-2H-benzo[h]chromene-2-carboxylate were negatively correlated with 1-(5-Phosphpribosyl)imidazole-4-acetate (*p* < 0.05). *Enterobacter* and cyclohexylammonium, dTDP-3-O-methyl-beta-L-rhamnose and 2-Hydroxy-2H-benzo[h]chromene-2-carboxylate were highly significantly correlated (*p* < 0.01); *Lentilactobacillus* showed a highly significant positive correlation with 3-Ketosucrose (*p* < 0.01) and *Limosilactobacillus* showed a highly significant positive correlation with 8-Methylthiooctyl glucosinolate (*p* < 0.01). However, *Lactiplantibacillus* and *Escherichia Shigella* were not correlated with differential metabolites.

Figure 9B shows the correlations between the differential metabolites of the AhGCK_vs_AhGLP comparison group. The results showed that 4-Fluorocyclohexadiene-cis,cis-1,2-diol-1-carboxylate and cyclohexylammonium were negatively correlated with the other differential metabolites (*p* < 0.05), 2-Hydroxy-2H-benzo[h]chromene-2-carboxylate was significantly correlated with each other (*p* > 0.05) but negatively correlated with the other differential metabolites (*p* < 0.05). *Enterobacter* was highly significantly correlated with 4-Fluorocyclohexadiene-cis,cis-1,2-diol-1-carboxylate, cyclohexylammonium and 2-Hydroxy-2H-benzo[h]chromene-2-carboxylate were highly significantly correlated (*p* < 0.01). Cyclohexylammonium was highly significantly correlated (*p* < 0.01) with *Lentilactobacillus* and *Leuconostoc*. 3-Ketosucrose was correlated with *Leuconostoc*, *unclassified Muribaculaceae*, and *Akkermansia* (*p* < 0.01).

Figure 9C shows the correlations among differential metabolites in the AhSCK_vs_AhSLP group. Findings indicate DIBOA, Pseudopelletierine, 2′-Deoxy-5-hydroxymethylcytidine-5′-diphosphate, UDP-kanosamine, and 5,6-EET were negatively correlated with 1,3E. Also, 5Z-Undecatriene and N-Acetylcadaverine were negatively correlated (*p* < 0.05). DIBOA and Pseudopelletierine were negatively correlated with 1-Piperidinecarboxaldehude and with each other (*p* < 0.05). 1-Piperidinecarboxaldehude and Sultopride were negatively correlated with 2′-Deoxy-5-hydroxymethylcytidine-5′-diphosphate, and UDP-kanosamine and 5,6-EET had significant negative correlations (*p* < 0.05). 5-(2-methoxyethy)isolongifal-5-ene was significantly negatively correlated with DIBOA, Pseudopelletierine, 2′-Deoxy-5-hydroxymethylcytidine-5′-diphosphate, UDP-Kanosamine, and 5,6-EET (*p* < 0.05). *Lentilactobacillus* was highly significantly correlated with sultopride, *Levilactobacillus* with 5,6-EET, and *Bacillus* with pseudopelletierine (*p* < 0.01).

Figure 9D shows the correlations between the differential metabolites in the AhGLP_vs_AhSLP comparison group. dTDP-3-O-methyl-beta-L-rhamnose showed significant negative correlations with the other nine differential metabolites (*p* < 0.05). *Lactiplantibacillus* was associated with dTDP-3-O-methyl-beta-L-rhamnose, amaranthin, medicagol, 5a,11a-Dehydrooxytetracycline, auraMycinone and 3-(2-Methylpropyl) pyridine were significantly correlated (*p* < 0.05). *Levilactobacillus* was significantly correlated with all the differential metabolites except Syringetin (*p* < 0.05). *Bacillus* was significantly correlated with dTDP-3-O-methyl-beta-L rhamnose and 3-Ketosucrose (*p* < 0.05).

## 4. Discussion

Moisture content is a key environmental factor affecting the silage fermentation process [22,23]. When the raw material moisture content is in the range of 60–72%, it is most conducive to the fermentation process dominated by LAB [24]. In this study, the moisture content of freshly cut amaranth was approximately 80%, which decreased to about 70% after wilting treatment. This moisture gradient provided a sound material basis for investigating the impact of moisture on silage fermentation, although it is important to recognize that wilting may also alter plant physiology and epiphytic microbiota independently of moisture reduction, potentially confounding the interpretation of a pure moisture effect.

The pH value of silage directly reflects fermentation quality, with a low pH environment effectively inhibiting the proliferation of undesirable microorganisms [25]. Generally, a pH value below 4.20 indicates good fermentation. In this study, the AhGCK had a pH value as high as 5.09, significantly exceeding the standard for high-quality silage. Although the pH value of AhSCK decreased, it still did not reach the high-quality standard of 4.20. However, after inoculation with *L. plantarum*, the pH values of silage under both moisture conditions dropped below 4.20, indicating that the addition of exogenous LAB effectively promoted the fermentation process. The decrease in pH is mainly attributed to the metabolic conversion of WSC by LAB. In an anaerobic environment, LAB proliferate rapidly and convert sugars into organic acids (primarily LA), leading to a decrease in system pH [23]. In this study, the LA content was significantly higher in the treatment groups inoculated with *L. plantarum*, which highly correlated with the trend of pH decrease. Notably, the pH value of AhGCK was as high as 5.09, indicating that LAB failed to become the dominant microbial community in this treatment, and the fermentation process was dominated by other microorganisms. This failure at 80% moisture without inoculation points to a possible threshold effect: above a certain moisture level, fast-growing Gram-negative bacteria may outcompete LAB even when fermentable carbohydrates are present, a hypothesis that warrants further testing.

The NH_3_-N content serves as a crucial indicator for assessing the extent of proteolysis in silage [26]. In the present study, the trend of NH_3_-N content exhibited an inverse correlation with CP content. Specifically, NH_3_-N content was lower in treatment groups inoculated with *L. plantarum* compared to non-inoculated groups, as well as in low-moisture treatment groups relative to high-moisture groups. This phenomenon can be attributed to the combined effects of LAB inoculation and moisture reduction: the rapid pH decline induced by LAB activity inhibits both proteolytic enzymes and protein-decomposing bacteria such as *Clostridium* spp. [27], while the low-moisture environment suppresses overall microbial metabolic activity through reduced water availability, thereby limiting protein deamination [28]. These findings are consistent with reports by He et al. and Kaewpila et al. [26,29], further validating the positive impact of LAB additives on silage protein preservation. Nevertheless, the relative contributions of plant-derived versus microbial proteases remain unknown, and the observed differences in NH_3_-N could also be influenced by wilting-induced changes in plant enzyme activity.

AA is the second most prevalent organic acid in silage, mainly originating from the metabolic activities of heterofermentative LAB [27]. In this study, compared with the AhGCK group, the AA content was higher in the other treatment groups, suggesting that wilting treatment and LAB inoculation enhanced the activity of heterofermentative LAB. PA and BA are generally considered unacceptable fermentation products in silage, as their production implies energy loss and a decline in fermentation quality. In this study, BA was not detected in any of the treatment groups, and the PA content was extremely low, indicating that extensive secondary fermentation did not occur during the fermentation process.

The extent of DM loss during fermentation is influenced by both the dominant microbial species and the availability of fermentable substrates. In this study, the DM and CP contents were lower in treatment groups not inoculated with LAB compared to inoculated groups, which aligns with reports by Yitbarek and Tamir, Irawan et al., and Zong et al. [30,31,32]. By rapidly establishing an acidic environment, LAB additives inhibit the activity of spoilage microorganisms, reducing the fermentation loss of nutrients, thereby demonstrating an advantage in preserving DM and CP.

The composition of structural carbohydrates in silage, particularly NDF and ADF content, serves as an important indicator for evaluating its rumen digestive characteristics. NDF primarily consists of cellulose, hemicellulose, and lignin. Excessively high NDF content can reduce feed digestibility, whereas lower NDF levels are beneficial for rumen fermentation [33]. In this study, under both moisture gradients, the NDF and ADF contents decreased to varying degrees following inoculation with *L. plantarum*. This phenomenon can be attributed to the acid hydrolysis of structural carbohydrates caused by the accumulation of organic acids resulting from exogenous LAB inoculation [34]. Research by Desta et al. confirmed that the acid hydrolysis process of structural carbohydrates is usually accompanied by the release of WSC [35].

WSC content is a critical limiting factor for microbial activity during the silage fermentation process, serving as the direct carbon and energy source for microbial metabolism [36,37]. During silage fermentation, WSC in the raw material not only acts as a fermentation substrate for LAB but can also be supplemented through the acid hydrolysis of fiber components. In this study, the WSC content was higher in the AhSLP than in AhSCK, indicating that the addition of *L. plantarum* effectively inhibited the consumption of WSC by competing microorganisms. Notably, regardless of whether LAB were inoculated or not, the WSC content of high-moisture silage was higher than that of low-moisture treatments. This may be because the overall microbial activity is higher in a high-moisture environment, releasing more bound carbohydrates through acid hydrolysis [38,39]. A higher residual WSC content has positive nutritional implications, as it can be rapidly fermented in the rumen to provide energy [35]. However, an alternative interpretation is also plausible: higher WSC in high-moisture silage might reflect lower microbial consumption due to substrate inhibition or preferential use of other carbon sources, and the underlying mechanisms remain unresolved.

Silage fermentation is essentially a microbially driven process, and the type and abundance of the participating microbial communities have a decisive impact on fermentation quality. Raw forage crops host a diverse epiphytic microbial community in the field. When environmental conditions change, microorganisms with better adaptability will occupy new ecological niches [28]. In this study, the coverage index for all samples was above 0.99, indicating that the sequencing depth adequately captured the majority of bacterial taxa present in the samples.

Analysis of diversity indices showed that after inoculating with *L. plantarum* under high-moisture conditions, the Shannon and Simpson indices decreased, indicating a reduction in bacterial diversity. Conversely, under low-moisture conditions, inoculation with *L. plantarum* led to an increase in these two indices. This result is not entirely consistent with findings reported by Wang et al. and Yang et al. [40,41]. Higher bacterial diversity typically reflects a complex microbial community structure, which in this study might be related to the coexistence of LAB and other potentially beneficial bacteria under low-moisture conditions. It is important to note, however, that diversity indices alone do not distinguish between beneficial diversity (e.g., coexistence of multiple LAB species) and spoilage-associated diversity (e.g., presence of enterobacteria). Consequently, the increased diversity observed in low-moisture inoculated silage should not be automatically interpreted as an improvement in quality.

At the phylum level, the predominant phyla across all treatment groups were Firmicutes, Proteobacteria, and Bacteroidota. After inoculation with *L. plantarum* under high-moisture conditions, the relative abundance of Firmicutes increased significantly, whereas that of Proteobacteria decreased significantly. In contrast, under low-moisture conditions, an opposite trend was observed. To gain a more in-depth understanding of this phenomenon, this study further analyzed the changes in the microbial community at the genus and species levels.

At the species level, in the high-moisture silage without lactic acid bacteria (LAB) inoculation, *Enterobacter cloacae* was the absolutely dominant species. After inoculation with *L. plantarum*, *L. plantarum* became the dominant species. This change is consistent with the findings of Guan et al. suggesting that exogenous LAB can effectively reshape the microbial community structure of high-moisture silage [42]. In AhSCK, *L. buchneri* was the dominant species. After inoculation with *L. plantarum*, *Levilactobacillus brevis* became the dominant species. This phenomenon implies that under low-moisture conditions, *Levilactobacillus brevis* may be more adaptable to the ecological niche of the stable fermentation phase compared to the exogenously added *L. plantarum* [43]. Notably, the relative abundances of *L. buchneri* and *L. plantarum*, which were the dominant species in the low-moisture silage, were largely unaffected by the exogenous additive, indicating that these two species exhibit good adaptability to the amaranth silage environment with a 70% moisture content.

Genera such as *Enterobacter* and *Clostridium* are typically regarded as undesirable microbial groups in silage. In this study, CP, DM, and AA contents were negatively correlated with *Enterobacter cloacae*, *Clostridium tyrobutyricum*, and *Lactococcus lactis*. *Enterobacter cloacae* and *Clostridium tyrobutyricum* compete with LAB for substrates, delaying the pH drop, leading to DM loss and NH_3_-N accumulation, thereby causing CP loss [44]. *Clostridium* spp. not only produce BA, reducing fermentation quality, but some species can also produce toxins harmful to animals [45]. *Lactococcus lactis*, as a facultatively heterofermentative LA bacterium, can metabolize pentoses via the phosphoketolase pathway to produce LA and AA [46]. The negative correlation observed in this study between *Lactococcus lactis* and DM, AA, and CP contents requires further investigation for validation, as there are currently few reports directly linking this bacterium to silage quality.

*L. buchneri* showed a highly significant positive correlation with CP, DM, and AA contents and a negative correlation with pH. As a heterofermentative LA bacterium, *L. buchneri* can convert LA to AA as a main metabolic product during fermentation [15] and metabolize arginine via the arginine deiminase pathway to produce ammonia, thereby alleviating self-inhibition under low pH conditions [47]. Moderate accumulation of AA has the potential to inhibit yeasts and molds, which can cause feed spoilage upon aerobic exposure. Therefore, heterofermentative LAB are valuable for reducing DM loss, improving aerobic stability, and minimizing CP loss [48]. This also explains the significant positive correlations observed between *Levilactobacillus brevis*, *Limosilactobacillus fermentum*, *unclassified Muribaculaceae*, and CP content.

*Unclassified Muribaculaceae*, belonging to the phylum Bacteroidota, have been reported as potentially beneficial bacteria in host-associated environments, although their ecological role in silage remains unclear. To date, no studies have characterized their function during ensiling. Research in other fields has shown that *Muribaculaceae* are associated with host health [49], extended lifespan in mice [50], and circadian rhythm oscillations in the rumen of dairy cows, with possible links to alleviating subclinical mastitis [51,52]. However, the relevance of these findings to silage fermentation is unknown. In the present study, the positive correlation between the relative abundance of this family and CP content was observed, which provides a preliminary clue for future investigations. Nevertheless, given the lack of direct evidence and the correlational nature of this finding, any functional interpretation regarding *Muribaculaceae* in the silage system should be treated with caution; it is possible that *Muribaculaceae* simply serve as an indicator of certain chemical conditions (e.g., nitrogen availability) rather than playing an active role in CP preservation.

In summary, the changes in fermentation parameters, nutritional components, and microbial communities observed in this study are the combined result of moisture content and exogenous LAB additive application. The high-moisture environment provided a competitive advantage for fast-growing Gram-negative bacteria like *Enterobacter*, leading to reduced fermentation quality. Wilting treatment inhibited competing microorganisms by reducing water activity, creating favorable conditions for LAB. The addition of exogenous *L. plantarum* further strengthened the competitive advantage of LAB, but the final outcome of microbial succession was significantly modulated by moisture content.

Notably, a close correspondence existed between the microbial community structure and fermentation parameters. The dominance of *Enterobacter cloacae* in the AhGCK treatment aligned closely with the characteristics of high pH, high NH_3_-N, and low LA content observed in this treatment. The enrichment of *L. buchneri* in the AhSCK treatment corresponded with its moderate fermentation quality. The dominance of the respective inoculated species in the *L. plantarum*-treated groups corresponded with the excellent fermentation parameters observed. This community-function correspondence confirms the functional roles of specific microbial taxa in silage fermentation.

To evaluate fermentation quality, numerous volatile organic acids in silage can be analyzed, including LA, AA, PA, and BA [53]. However, more complex compounds can be produced by different microbial populations in natural or LAB-inoculated silage. Clearly, the restricted detection of numerous organic acids in silage cannot fully represent its metabolomic changes. Metabolomics techniques can more accurately represent the metabolite composition in the environment and have also been applied in silage evaluation [19,54], thereby revealing the patterns of metabolite variation across different biological samples. Metabolites are the end products of cellular regulatory processes, and their levels can be viewed as the biological system’s response to genetic and environmental changes.

This study employed an untargeted metabolomics approach to systematically investigate changes in the metabolite profile of amaranth silage under different treatment conditions. Through identification and comparison of 497 metabolites across four treatment groups, significant metabolic differences were observed among groups, and these differences were closely correlated with specific microbial taxa. This metabolite-microbe association reveals the complex biochemical interaction network during amaranth ensiling, providing new insights into the microbial metabolic regulatory mechanisms underlying silage fermentation.

PCA results showed that AhSCK was distinctly separated from the other three treatment groups on the score plot, indicating that the ensiling process itself induced substantial metabolite reorganization, while additive treatment further altered metabolite accumulation patterns. Notably, the separation between AhGCK and AhSCK demonstrated that moisture content of the raw material exerts a decisive impact on the silage metabolome. Similarly, Wang et al. reported in alfalfa silage with different moisture contents that wilting significantly altered the microbial community structure, markedly increasing the relative abundance of LAB, whereas high-moisture treatment promoted the proliferation of non-lactic acid bacteria such as *Enterobacter* [55]. The dominant microorganism in AhGCK was *Enterobacter cloacae*, while AhSCK was dominated by *L. buchneri* and *Levilactobacillus brevis*. This difference in microbial communities is necessarily reflected in the metabolite profiles, as different microorganisms possess distinct metabolic networks and product spectra [28].

The tight clustering of QC samples in this study confirmed the high stability of the analytical platform and data reliability. The R^2^Y (>0.99) and Q^2^ (>0.97) values of the OPLS-DA models were both close to 1, indicating excellent fit and predictive ability of the constructed discriminant models. Ke et al. also reported similar model parameters (R^2^Y > 0.98, Q^2^ > 0.95) in alfalfa silage research, further validating the reliability of multi-omics integrated analysis for elucidating silage fermentation mechanisms [56].

The substantial differences in the number of differentially abundant metabolites (DAMs) among comparison groups (1152 vs. 1105 vs. 512 vs. 761) reflect the varying intensity with which different treatment factors regulate the metabolic network. The AhGCK_vs_AhSCK comparison yielded up to 1152 DAMs, with 1047 up-regulated, indicating that changes in moisture content exerted the most dramatic impact on metabolite accumulation. This phenomenon can be attributed to the combined effects of a high-moisture environment: on one hand, it preserves plant enzyme activity, allowing sustained plant metabolic activity during the early stage of ensiling [27]; on the other hand, it promotes the proliferation of Gram-negative bacteria such as *Enterobacter*, which possess complex secondary metabolic capabilities and can generate a diverse array of metabolites [2].

KEGG enrichment analysis revealed that DAMs from different comparison groups were enriched in distinct metabolic pathways, reflecting the specific regulation of the metabolic network of the amaranth-microbe interaction system by different treatment conditions. In the AhGCK_vs_AhSCK comparison group, DAMs were primarily enriched in secondary metabolic pathways such as diterpenoid biosynthesis and isoquinoline alkaloid biosynthesis. Diterpenoid compounds in plants typically serve as defensive secondary metabolites, induced upon mechanical damage or microbial invasion [57]. Physical operations during ensiling, such as chopping and compaction, simulate mechanical damage signals, thereby activating diterpenoid biosynthesis. Copeland et al. also found in rice that mechanical damage rapidly activated the expression of genes involved in defensive diterpenoid synthesis within 15 min [58]. The enrichment of the isoquinoline alkaloid biosynthesis pathway is closely related to microbial interactions; many members of this compound class (e.g., sanguinarine, chelerythrine) possess significant antimicrobial activity [59], potentially reflecting a plant chemical defense response against invasion by opportunistic pathogens such as *Enterobacter* during the early ensiling stage. Li et al. found that lactic acid bacteria inoculation significantly increased the abundance of terpenoids (25.29%) and esters (17.08%), confirming that secondary metabolites are important mediators through which microorganisms regulate silage quality [60].

In the AhGCK_vs_AhGLP comparison group, DAMs were enriched in pathways including isoquinoline alkaloid biosynthesis, monobactam antibiotic biosynthesis, and indole alkaloid biosynthesis. This enrichment pattern suggests that exogenous LAB inoculation may induce reprogramming of plant secondary metabolism. Monobactam compounds (e.g., nocardicins, sulfazecin) are a class of secondary metabolites with antimicrobial activity [61], and activation of their biosynthesis could reflect bidirectional chemical dialog in plant-probiotic interactions. Zhang et al. confirmed that inoculation with *L. plantarum* significantly altered the metabolite profile of alfalfa silage, particularly at the level of amino acid and energy metabolism pathways, even though conventional fermentation parameters (pH, organic acids) might not show significant improvement [62]. This suggests that metabolomic analysis can capture more sensitive signals of plant-microbe interactions than traditional fermentation indices.

DAMs in the AhSCK_vs_AhSLP comparison group were mainly enriched in pathways such as unsaturated fatty acid biosynthesis, linoleic acid metabolism, and diterpenoid biosynthesis. Unsaturated fatty acids play important roles in silage, not only as an energy source but also in regulating cell membrane fluidity and signal transduction processes [62]. Yang et al. found in whole-plant corn silage that lactic acid bacteria inoculation significantly altered the accumulation pattern of fatty acid metabolites, with enrichment of the linoleic acid metabolism pathway closely related to silage aerobic stability [63]. The promotion of unsaturated fatty acid accumulation by lactic acid bacteria inoculation may be achieved through two mechanisms: contribution from the fatty acid synthesis metabolism of the lactic acid bacteria themselves and alteration of environmental pH by lactic acid bacteria metabolites (e.g., organic acids), which activates plant-derived lipase activity and promotes the release of ester-bound fatty acids [4].

In the AhGLP_vs_AhSLP comparison group, DAMs were enriched in pathways including purine metabolism, cyanoamino acid metabolism, histidine metabolism, and polyketide sugar unit biosynthesis. This comparison group reveals the impact of the ensiling process itself (rather than the additive) on metabolites, with enriched pathways primarily related to energy metabolism and nitrogen metabolism. Enrichment of purine metabolism reflects degradation and reorganization of nucleic acids during ensiling, an inevitable consequence of nucleotide turnover during microbial growth and reproduction [64]. Kilstrup et al. reported that lactic acid bacteria require a continuous supply of purines and pyrimidines during growth, and their metabolic state directly influences fermentation product composition [65]. The enrichment of the cyanoamino acid metabolism pathway deserves special attention because cyanogenic glycosides in certain plants can act as potential anti-nutritional factors [66], and the safety of their degradation products (e.g., hydrogen cyanide) is a key concern in silage research.

Through correlation analysis between the top 10 DAMs from each comparison group and differential microbial genera, multiple highly statistically significant metabolite-microbe association pairs were identified. These associations provide direct evidence for understanding how microorganisms regulate metabolite accumulation.

In the AhGCK_vs_AhSCK comparison group, *Enterobacter* showed highly significant positive correlations with cyclohexylammonium, dTDP-3-O-methyl-β-L-rhamnose, and 2-hydroxy-2H-benzo[h]chromene-2-carboxylate. The genus *Enterobacter* includes various Gram-negative bacteria with active metabolic capabilities; some members can utilize diverse carbon sources and produce complex secondary metabolites [44]. In the present study, *Enterobacter* abundance was positively correlated with these metabolites, consistent with the possibility that this genus contributes to their accumulation, but this remains to be tested [67]. The positive correlations observed in this study indicate that *Enterobacter* is a candidate microbe associated with these metabolites; however, further experiments are required to establish causality. Notably, compounds such as cyclohexylammonium can serve as nitrogen storage forms in the metabolism of certain microorganisms [68]; their accumulation may reflect the nitrogen metabolism characteristics dominated by *Enterobacter*. Conversely, the genus *Lentilactobacillus* showed a highly significant positive correlation with 3-ketosucrose, an oxidized derivative of sucrose that can serve as a precursor for exopolysaccharide synthesis in the metabolism of some lactic acid bacteria (particularly *L. buchneri*) or participate in the modification of antimicrobial substances such as bacteriocins [46]. The highly significant positive correlation between the genus *Limosilactobacillus* and 8-methylthiooctyl glucosinolate is also noteworthy. Glucosinolates and their degradation products (isothiocyanates, thiocyanates, etc.) possess antioxidant and antimicrobial activities and may serve as important mediator molecules in lactic acid bacteria-plant interactions [69].

Analysis of the AhGCK_vs_AhGLP comparison group revealed that *Enterobacter* was highly significantly correlated with 4-fluorocyclohexadiene derivatives, cyclohexylammonium, and benzochromene derivatives. This pattern is similar to that in the AhGCK_vs_AhSCK comparison group, suggesting a potential association of *Enterobacter* with the presence of the aforementioned metabolites; however, correlation does not imply causation, and further experiments (e.g., pure culture inoculation or genetic manipulation) are required to determine whether *Enterobacter* directly contributes to their accumulation. Of particular note, cyclohexylammonium showed highly significant correlations with *Lentilactobacillus* and *Leuconostoc*, while 3-ketosucrose was highly significantly correlated with *Leuconostoc*, unclassified *Muribaculaceae*, and *Akkermansia*. *Akkermansia* has garnered significant attention as a next-generation probiotic candidate, playing an important role in maintaining intestinal mucus layer integrity [70]. Its detection in silage and correlation with specific metabolites suggest a possible link that warrants further research, but no functional role can be inferred from correlation alone.

In the AhSCK_vs_AhSLP comparison group, *Lentilactobacillus* showed a highly significant correlation with sultopride, *Levilactobacillus* with 5,6-EET, and *Bacillus* with pseudopelletierine. Sultopride is an aromatic compound whose biological function remains unclear, but its correlation with *Lentilactobacillus* may be associated with the presence of *Lentilactobacillus*, but this hypothesis requires experimental validation. 5,6-EET (5,6-epoxyeicosatrienoic acid) is a cytochrome P450 metabolite of arachidonic acid, known for its vasoregulatory and anti-inflammatory functions in mammals [71]; its presence in silage and correlation with *Levilactobacillus* represents a novel finding warranting further investigation. Xu et al. found in alfalfa silage that inoculation with different lactic acid bacteria strains significantly altered the metabolite profile, with specific metabolites showing strong correlations with particular bacterial genera, consistent with the patterns observed in this study [72]. In the AhGLP_vs_AhSLP comparison group, *Lactiplantibacillus* showed significant correlations with multiple metabolites, including dTDP-3-O-methyl-β-L-rhamnose, amaranthin, medicagol, and deoxytertonine. Amaranthin is a betalain pigment specific to amaranth with strong antioxidant activity [73]; its correlation with *Lactiplantibacillus* suggests that lactic acid bacteria may influence the stability or modification state of such pigments by altering the microenvironmental pH or producing glycosyltransferases. Medicagol is a coumestan phytoestrogen commonly found in leguminous plants such as alfalfa [74]; its presence in amaranth and correlation with lactic acid bacteria provides a new perspective for understanding the physiologically active functions of silage.

In summary, the metabolite differences and metabolite-microbe correlations observed in this study reflect the complex interaction network within the silage ecosystem. Moisture content and LAB additives regulate microbial community structure, thereby reshaping the overall metabolite profile of the system. From an ecological perspective, the high-moisture environment of the AhGCK treatment group provided a competitive advantage for fast-growing Gram-negative bacteria such as *Enterobacter*. These microorganisms possess diverse secondary metabolic capabilities, enabling them to produce various metabolites such as cyclohexylammonium and benzochromene derivatives. Their metabolic activities consume water-soluble carbohydrates and produce alkaline substances such as ammonia, which are not conducive to rapid pH decline [75]. In contrast, the wilting treatment in the AhSCK group reduced water activity, creating osmotic stress for moisture-sensitive microorganisms such as *Enterobacter*, thereby providing a competitive advantage for lactic acid bacteria, particularly *L. buchneri* and *L. brevis* [48]. The metabolism of these lactic acid bacteria is relatively focused on glycolysis and organic acid production, resulting in a comparatively simplified metabolite profile, reflected by a lower number of DAMs (512) and a more balanced up/down ratio. Ke et al. also found significant differences in metabolite profiles between high-moisture and low-moisture alfalfa silage, and these differences were modulated by lactic acid bacteria inoculation [56].

The regulation of the metabolite profile by LAB inoculation exhibited strain specificity and moisture dependence [9]. Under high-moisture conditions, *L. plantarum* inoculation partially inhibited the activity of *Enterobacter* but did not completely alter the microbial community structure; the metabolite profile still retained many characteristics associated with *Enterobacter* (e.g., the presence of cyclohexylammonium metabolites). Under wilted conditions, *L. plantarum* inoculation strengthened the competitive advantage of lactic acid bacteria, and the metabolite profile became more characteristic of a “LAB type,” enriched in pathways closely related to LAB metabolism, such as unsaturated fatty acid biosynthesis and linoleic acid metabolism [76].

Notably, certain metabolites (e.g., dTDP-3-O-methyl-β-L-rhamnose) were identified as DAMs in multiple comparison groups and showed significant correlations with several genera, suggesting that such compounds may act as “hub metabolites” in silage fermentation, playing similar functional roles under different treatment conditions. Yang et al. found in whole-plant corn silage that D-galacturonic acid could serve as a biomarker for clostridial overgrowth, showing a strong positive correlation (R^2^ = 0.87, *p* < 0.01) with the abundance of *Clostridium beijerinckii* [7]. The dTDP-rhamnose derivative identified in this study may also possess similar biomarker potential, warranting further validation.

Given that metabolomics provides a more comprehensive and sensitive dataset than traditional fermentation parameters, it serves as a valuable complement for evaluating silage quality and microbial metabolic activity [3]. This approach not only reveals specific metabolite-microbe associations that establish a theoretical basis for developing rapid, metabolite-based diagnostic tools, but also yields critical insights into the underlying interaction networks. Based on these insights, more precise multi-species inoculant strategies can be designed, for example by targeting specific metabolic pathways to direct and optimize the fermentation process.

However, this study has certain limitations. Untargeted metabolomics comprehensively captures changes in the metabolite profile, but structural identification of some metabolites may lack precision; correlation analysis reveals associations between metabolites and microbes but cannot establish causality; findings from in vitro static culture conditions require further validation through in vivo animal studies. Future research could combine stable isotope tracing, in vitro pure culture techniques, and animal trials to deeply dissect the molecular mechanisms of key metabolite-microbe interaction pairs, providing a more robust theoretical basis for the efficient utilization of amaranth silage.

## 5. Conclusions

Moisture content is the primary ecological determinant in amaranth silage, dictating whether *Enterobacteria* or lactic acid bacteria dominate the fermentation. Reducing moisture from 80% to 70% suppresses spoilage organisms and creates a niche that favors moisture-adapted *Lentilactobacillus buchneri* and *Levilactobacillus brevis*. Exogenous *L. plantarum* accelerates fermentation but its success depends on moisture: it outcompetes others at 80% moisture, yet is partially displaced by native *L. brevis* at 70% moisture. Metabolomics confirms moisture as the main driver of metabolome remodeling—high moisture activates plant defense pathways, while LAB inoculation shifts metabolism toward fatty acids and antioxidants. Strong microbe-metabolite correlations offer candidate biomarkers for quality monitoring, and provide a theoretical foundation for developing moisture-targeted ensiling strategies, thereby enhancing fermentation stability and nutritive value.

## Figures and Tables

**Table 1:** Chemical composition and microbial counts of pre-ensiled amaranth at different moisture contents.

Items	GFM (80%)	SFM (70%)
DM (%FM)	21.00 ± 1.23 b	32.03 ± 0.16 a
CP (%DM)	6.94 ± 0.06 a	7.99 ± 0.85 a
NDF (%DM)	46.37 ± 2.68 a	47.39 ± 0.93 a
ADF (%DM)	33.22 ± 1.05 a	36.70 ± 0.74 a
WSC (%DM)	1.18 ± 0.05 a	1.35 ± 0.28 a
LAB (Log_10_ cfu/g FM)	3.84 ± 0.55 a	3.90 ± 0.78 a
Coliform bacteria (Log_10_ cfu/g FM)	2.73 ± 2.53 a	4.65 ± 2.04 a
Aerobic bacteria (Log_10_ cfu/g FM)	6.45 ± 0.73 a	4.03 ± 2.15 b
Yeast (Log_10_ cfu/g FM)	4.55 ± 2.58 a	5.26 ± 0.37 a
Mold (Log_10_ cfu/g FM)	2.00 ± 1.76 a	0.90 ± 0.39 a

Note: Data are presented as mean ± standard deviation. Different lowercase letters within the same row indicate significant differences (*p* < 0.05). Abbreviations: FM, fresh matter; DM, dry matter; CP, crude protein; NDF, neutral detergent fiber; ADF, acid detergent fiber; WSC, water-soluble carbohydrates; LAB, lactic acid bacteria.

**Table 2:** Chemical composition and fermentation quality of amaranth silage after 60 days of ensiling.

Items	80%		70%		SEM	Significance		
	AhGCK	AhGLP	AhSCK	AhSLP		M	A	M × A
DM (%FM)	20.90 ± 1.39 Ab	21.13 ± 0.16 Ab	31.46 ± 0.72 Aa	31.37 ± 0.51 Aa	3.0026	<0.0001	0.9096	0.8023
CP (%DM)	5.90 ± 0.69 Ab	6.15 ± 0.26 Ab	7.92 ± 0.85 Aa	8.59 ± 0.75 Aa	0.6601	0.0006	0.2630	0.6318
NDF (%DM)	48.31 ± 2.19 Aa	44.83 ± 2.69 Aa	46.44 ± 1.94 Aa	46.17 ± 0.74 Aa	0.7166	0.8321	0.1683	0.2276
ADF (%DM)	39.37 ± 1.18 Aa	36.09 ± 1.58 Aa	39.23 ± 1.29 Aa	38.52 ± 1.59 Aa	0.7606	0.0848	0.0620	0.0543
WSC (%DM)	0.37 ± 0.10 Aa	0.31 ± 0.09 Aa	0.16 ± 0.03 Aa	0.25 ± 0.06 Aa	0.0448	0.1046	0.0503	1.0000
pH	5.09 ± 0.39 Aa	4.16 ± 0.05 Bb	4.51 ± 0.04 Aa	4.20 ± 0.01 Ba	0.2147	0.0790	0.0021	0.0244
LA (%FM)	1.42 ± 0.20 Bb	3.65 ± 0.12 Ab	3.85 ± 0.03 Ba	4.46 ± 0.09 Aa	0.6644	<0.0001	<0.0001	<0.0001
AA (%FM)	0.37 ± 0.10 Ab	0.40 ± 0.09 Aa	0.51 ± 0.04 Aa	0.49 ± 0.04 Aa	0.0340	0.0077	0.8659	0.3564
PA (%FM)	0.35 ± 0.13 Aa	0.12 ± 0.02 Ba	0.23 ± 0.14 Aa	0.18 ± 0.06 Aa	0.0488	0.5262	0.0537	0.1210
NH_3_-N/TN	1.42 ± 0.32 Aa	1.02 ± 0.03 Aa	1.17 ± 0.08 Aa	1.04 ± 0.22 Aa	0.0920	0.3018	0.0503	0.2679

Note: Data are presented as mean ± standard deviation. Different uppercase letters indicate significant differences between moisture treatments within the same additive group (*p* < 0.05); different lowercase letters indicate significant differences between additive treatments within the same moisture group (*p* < 0.05). SEM, standard error of the mean; M, moisture effect; A, additive effect; M × A, interaction effect; Abbreviations are as listed in Table 1, with the following additions: LA, lactic acid; AA, acetic acid; PA, propionic acid; NH_3_-N/TN, ammonia nitrogen to total nitrogen ratio.

**Table 3:** Alpha diversity indices of bacterial communities in amaranth silage after 60 days of ensiling.

Items	80%		70%		SEM	Significance		
	AhGCK	AhGLP	AhSCK	AhSLP		M	A	M × A
ACE	227.2960 Aa	338.4793 Aa	399.6387 Aa	252.3845 Aa	39.6678	0.3947	0.7191	0.0191
Chao1	123.4017 Bb	276.1481 Aa	262.6909 Aa	237.1075 Aa	34.7676	0.1825	0.0965	0.0252
Simpson	0.6134 Aa	0.6056 Aa	0.4089 Ba	0.7776 Aa	0.0754	0.8584	0.0625	0.0532
Shannon	1.9150 Aa	2.4871 Aa	1.8603 Ba	3.5092 Aa	0.3826	0.2721	0.0194	0.2238
Coverage	0.9972 Aa	0.9919 Ba	0.9928 Ab	0.9943 Aa	0.0012	0.4952	0.2106	0.0370

Note: Data are presented as mean ± standard deviation. Different uppercase letters indicate significant differences between moisture levels (80% vs. 70%) within the same additive treatment (*p* < 0.05); different lowercase letters indicate significant differences between additive treatments (control vs. *L. plantarum*) within the same moisture level (*p* < 0.05). SEM, standard error of the mean; M, moisture effect; A, additive effect; M × A, interaction effect; Coverage values > 0.99 indicate adequate sequencing depth for all samples.

## Data Availability

The 16S rRNA gene sequences of the amaranth silage samples were deposited in the NCBI database with the accession number PRJNA888204.
